# In-person school reopening and the spread of SARS-CoV-2 during the second wave in Spain

**DOI:** 10.3389/fpubh.2022.990277

**Published:** 2022-10-13

**Authors:** Raül Tormos, Pau Fonseca i Casas, Josep Maria Garcia-Alamino

**Affiliations:** ^1^Centre d'Estudis d'Opinió - Generalitat de Catalunya, Barcelona, Spain; ^2^Department of Law and Political Science, Open University of Catalonia, Barcelona, Spain; ^3^Universitat Politecnica de Catalunya, Barcelona, Spain; ^4^Global Health, Gender and Society, Open University of Catalonia, Barcelona, Spain

**Keywords:** COVID-19, SARS-CoV-2, in-person school reopening, non-pharmaceutical intervention, interrupted time-series analysis

## Abstract

We investigate the effects of school reopening on the evolution of COVID-19 infections during the second wave in Spain studying both regional and age-group variation within an interrupted time-series design. Spain's 17 Autonomous Communities reopened schools at different moments in time during September 2020. We find that in-person school reopening correlates with a burst in infections in almost all those regions. Data from Spanish regions gives a further leverage: in some cases, pre-secondary and secondary education started at different dates. The analysis of those cases does not allow to conclude whether reopening one educational stage had an overall stronger impact than the other. To provide a plausible mechanism connecting school reopening with the burst in contagion, we study the Catalan case in more detail, scrutinizing the interrupted time-series patterns of infections among age-groups and the possible connections between them. The stark and sudden increase in contagion among older children (10–19) just after in-person school reopening appears to drag the evolution of other age-groups according to Granger causality. This might be taken as an indirect indication of household transmission from offspring to parents with important societal implications for the aggregate dynamics of infections.

## Highlights

- Interrupted time-series analyses show that in-person school reopening precedes and correlates with a posterior growth in contagion in almost all Spanish regions that reopened at different moments in time during September 2020 in Spain.
- A more granular analysis of the dynamics of age-groups in the Spanish region of Catalonia indicates that infections among individuals aged 10–19 grew earlier and faster than the rest just after school-reopening, driving the evolution of other age-groups in a Granger causal process.

## Introduction

In many countries, several non-pharmacological interventions (NPIs) to mitigate the spread of SARS-CoV-2 in the community have been tested and proved effective, such as online schooling, mandatory mask wearing or the closure of bars and restaurants (1–8). Some published studies focus on specific factors, like age (9–11), while others define more general models to identify the effects of non-pharmacological interventions (6), or the effects of the vaccination process on the population (12). A study of the pandemic situation in Catalonia proposes the use of a *Digital Twin* (13). In that study, a combination of a simulation and an optimization model through a continuous validation process allows understanding the effects of the different NPIs on the population by analyzing the change points brought about by new cases. Similarly, a study applying Bayesian inference to a type of epidemiological SIR model (14) analyzes the change points and infers the effects of different interventions on the evolution of new cases.

During the first wave of the pandemic in Spain (47.3 M inhabitants), and as a part of the lockdown, in-person schooling was shut down. Nonetheless, both the effectiveness and social consequences of in-person school closure remain a controversial issue. Different studies employing various approaches, for school facilities (15–21) and specifically for child care facilities (22), have tried to discern if schools are a vector for the propagation of the infection and whether children have an impact on that spread (23). Two other studies with a similar focus to ours at trying to estimate the effect of in-person school reopening arrived at opposing conclusions when analyzing the second pandemic wave in Italy: one found a link between school reopening and the resurgence of the virus (24), while the other did not (25). For other similar diseases, like influenza outbreaks, closure of in-person schooling has been an effective non-pharmaceutical intervention (26, 27). Being the spread of SARS-CoV-2 mainly airborne (28–30), knowing that to talk increases the transmission risk (31), and that the risk raises in poorly ventilated environments (32), it seems plausible that online schooling will reduce community transmission as compared to in-person schooling. Besides that, children seem to have equivalent nasopharyngeal viral loads to adults (16, 33–35), even though the youngest (ages 0–10) may have had lower susceptibility (36) therefore some studies suggest that the transmission is mainly in households (37), although other suggest that although they have lower susceptibility, the youngest ones are more infectious than older individuals (38). Therefore, the spread on schools would remain high if limited measures are applied to mitigate transmission (35, 39). These different evidences lead to the definition of several official advices and reports with the purpose to lessen viral outbreaks in schools in the context of in-person schooling (40, 41) with special focus on the Accumulated Incidence (AI) in the community.

In this paper we analyze the role of in-person school reopening in Spain on the evolution of infections. Using an interrupted time-series perspective, we explore and model the dynamics followed by the different Spanish regions, Autonomous Communities, that reopened schools at varying moments in time during September 2020. The impact of school reopening is understood in the models as an external shock or interruption to the series. The evidence points to a correlation between school reopening and a posterior outbreak in contagion across most ACs. We further provide a plausible causal mechanism for that association by studying the Catalan situation in more detail. For this case, we analyze actual data on the evolution of infections among the different age-groups and their interconnected dynamics, identifying some key sociological patterns. A sudden burst in contagions among school-age individuals (10–19) takes place just after in-person school reopening and appears to drag the dynamics of other age-groups. We argue that actual data may contain age-dependent measurement error. Therefore, we replicate our interrupted time-series analysis using corrections for measurement bias as a robustness test. We weight the actual data by the levels of prevalence by age-group as obtained from large-scale probability sample surveys (42–45). This reanalysis confirms our main findings, what constitutes a strong robustness test, and offers further light into additional phenomena overlooked in the official incidence rate records. The data sources we use are provided by the Open Data service of the Catalonia regional government (46), accessed through the Socrata connector (47), and the National Statistics Institute INE (48).

## School reopening across Spanish regions

**Figure 1:**
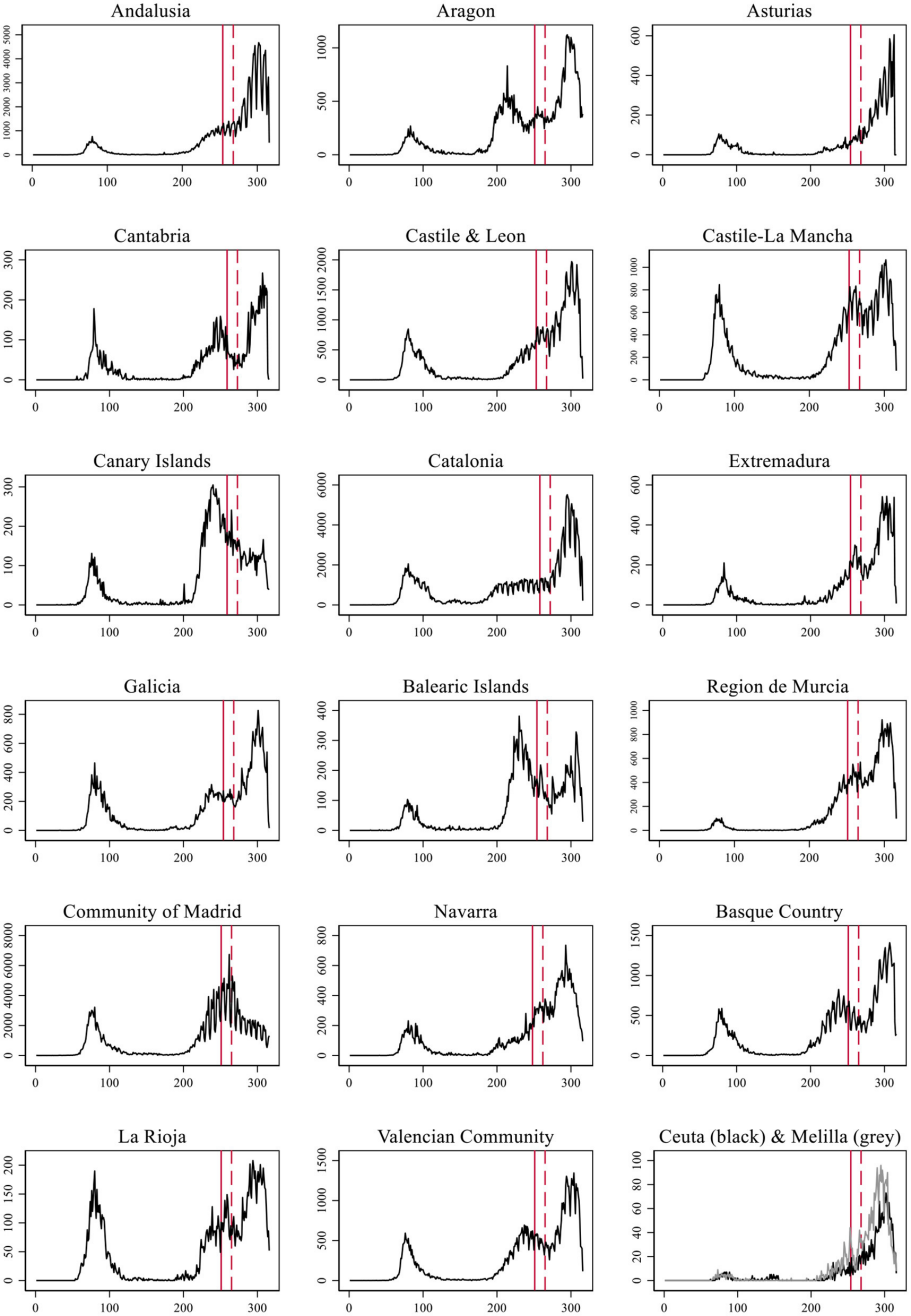
Evolution of the number of COVID-19 cases per day in each Autonomous Community of Spain.

In Figure 1 we present the number of daily COVID-19 cases detected in each Autonomous Community (AC, from now on) from the 1st of January until November 11th of 2020 (49). The date of school reopening is indicated with a vertical red line, and a dotted red line shows the 14th day after reopening. This range of time corresponds to the most likely incubation period for a child who contracted the virus on the 1st day of reopening and used as the official quarantine period. Schools were scheduled to open in different dates during September depending on what the government of each AC had arranged. In the cases in which pre-secondary and secondary education did not start the same day, we used the opening of secondary education as older children are assumed to have a stronger capacity to infect others. The figure includes the cases of Ceuta and Melilla which are Spanish autonomous cities in the North of Africa.

In all but two cases we observe an exponential growth in contagion 14 days after school reopened in September. The two exceptions are the Madrid Community and the Canary Islands. In these two cases, the peak of the second wave occurred before school reopening and containment measures were already applied previously. In the remaining 15 cases along with the two autonomous cities, the pattern is of an exponential growth. In six ACs the upsurge came after the second wave was being successfully contained, leading to a third wave: Cantabria, Castile-La Mancha, Extremadura, Balearic Islands, Basque Country, Region of Murcia, Navarra, Rioja, and Valencian Community. In four cases, the exponential growth came after a stationary situation: Andalusia, Aragon, Catalonia, and Galicia. In the four remaining cases, the number of daily contagions was already increasing before, but school reopening established the point where it definitively bursted.

Next, we perform a set of interrupted time-series Poisson regression models corrected for over dispersion, one for each AC, using the incidence rate as a dependent variable and having as predictors a linear trend (time) and a dummy variable representing an external shock to the series: in-person school reopening (the intervention), where 1 is the time-period with in-person classes and 0 otherwise. Therefore, in the models, the incidence rate (*r*) is assumed to be given by:


(1) $$\begin{array}{l} r{=\log}^{-1}\left(\alpha_{0}+\beta_{1}\left(time\right)+\beta_{2}\left(intervention\right)\right)\\ =e^{\alpha_{0}+\beta_{1}\left(time\right)+\beta_{2}\left(intervention\right)} \end{array}$$

**Figure 2:**
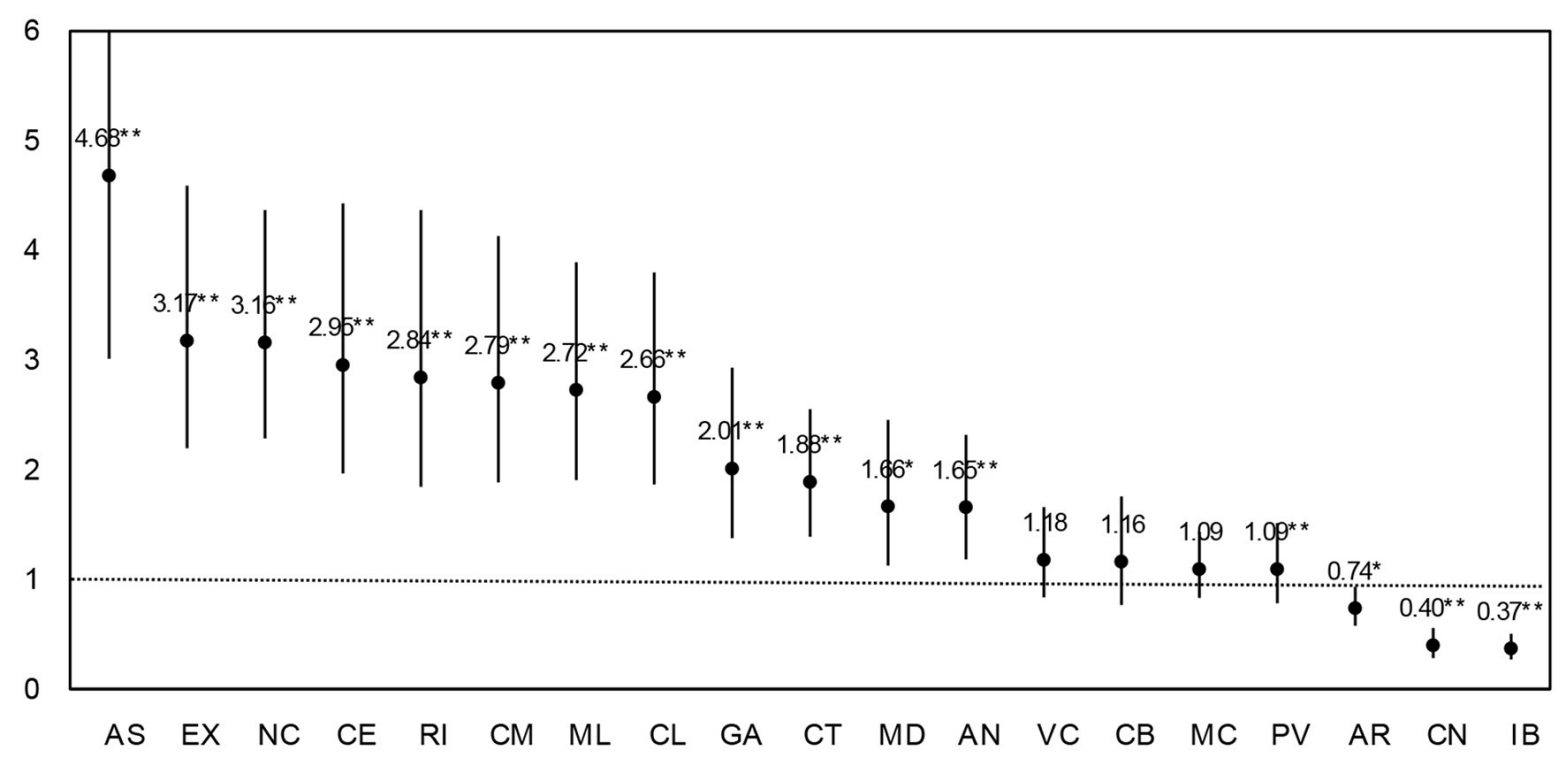
Effect of school reopening in each ACs. Estimates are incidence-rate ratios from Poisson regressions corrected for over dispersion. 95% CI. **p < 0.01, *p < 0.05.

Full results of those regressions are presented in Supplementary Table 1. According to this modeling strategy and looking at the incidence-rate ratios (the change in the incidence rate due to the intervention) in Figure 2, school reopening implied a clear raise in the risk of contagion for the general population in all but three ACs (84% of ACs). The exceptions were Aragon (AR), Cantabria (CN), and the Balearic Islands (IB). Asturias was the most affected AC. Reopening face-to-face classes increased 4.7 times the rate of infection in this region as compared to the period when schools were closed. In seven other ACs the rate of infection tripled (or almost) after the reopening: Extremadura, Navarra, Ceuta, Rioja, Castile-La Mancha, Melilla, and Castile and Leon. In other four cases the rate doubled or nearly: Galicia, Catalonia, Madrid, and Andalusia. In the remaining four ACs, the impact of reopening was still relevant implying an increase in cases in between 20 and 10% (Valencian Community, Cantabria, Murcia, and the Basque Country).

Next, we provide an estimate of the average effect of school reopening across ACs (*r*_*ij*_) by using the pooled dataset of all ACs. We employ a panel data approach that conveniently accounts for the clustering of cases in geographical units (see Table 1). We run a random effects Poisson regression considering entity-specific intercepts for Autonomous Communities. The model contains a dummy variable for school reopening and a time trend, as shown in the following equation:


(2) $$\begin{array}{l} r_{it}=e^{\alpha_{0}+\beta_{1}\left(time_{it}\right)+\beta_{2}\left(intervention_{it}\right)+u_{i}} \end{array}$$

For *i* = 1, …, 19 ACs and *t* = 1, …, 320 days observed. The random effects *u*_*i*_ are assumed to be normally distributed with mean 0 and variance $\sigma_{u}^{2}$.

**Table 1:** Random effects Poisson regressions with entity-specific intercepts (ACs).

	**IRRs**
School reopening	1.760***
	(0.005)
Time (linear trend)	1.009***
	(0.000)
Constant	36.167***
	−8.699
Ln Alpha	0.15
Alpha	1.162
Log likelihood	−538,910.76
Observations	5,966

The estimates of Poisson models are incidence-rate ratios (IRRs).

Standard errors are in parentheses.

^***^p < 0.01,

^**^p < 0.05,

^*^p < 0.1.

On average, school reopening has a sizeable and significant statistical effect. Estimates for school reopening indicate a robust mean impact of school reopening on the incidence of daily infections across ACs^1^. The incidence-rate ratio associated to school reopening implies a 76% average increase in risk for the population of becoming infected when comparing before and after reopening. Of course, with observational evidence, third variables temporally coinciding with school reopening, such as the return to work, could confound the association. Correlation does not imply causation, but neither precludes it.

**Figure 3:**
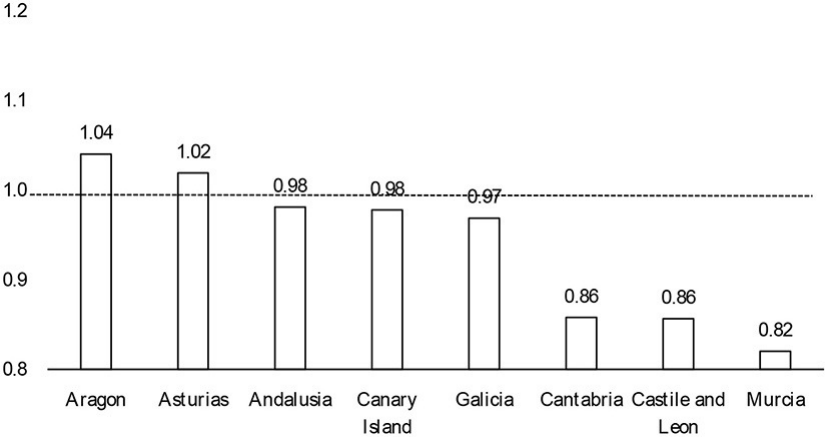
Ratio of the incidence-rate ratios of opening secondary education with respect to pre-secondary.

Secondary and pre-secondary education (kindergarten and primary school) started at different moments in time in eight ACs. We can benefit from these naturally occurring phenomenon and use it to test whether the opening of any of the two educational stages had a stronger aggregate impact than the other. We run two separate Poisson regression models for each of these eight ACs, one using time and the date of pre-secondary education reopening as a predictor variable and the other employing the date of secondary education reopening instead. Detailed results are shown in Supplementary Table 3. In Figure 3, we present the ratio of the effects of opening secondary education with respect to pre-secondary (the ratio of the incidence rate ratio). In five out of eight cases (63%), there are almost no differences between coefficients. In the remaining three cases the opening of pre-secondary education had a stronger impact than the start of secondary education.

## Indirect evidence of household transmission

To gain further insight as to which mechanisms may drive the outbreak of infections coinciding with in-person school reopening, we study the Catalan case with more detailed data on age-groups (46). We explore the rate of infections per day within each age-group. Our hypothesis is that contagion inside family units with children might have been crucially boosted due to the school reopening. In aggregate terms, the return to in-person classes would have fostered a silent spread of the virus through the community with visible societal consequences 2 weeks later. Lacking direct measures on family units, we study the aggregate dynamics of infection in age-groups that might be involved. Individuals in their forties (40–49) are more likely to have children between the ages of 10 and 19 and live together with them (50). Using aggregate time series data, in the following analysis we show how these two age groups evolve similarly over time during the second wave of the pandemic, and that school reopening might be one main driver of the exponential growth in infections among children aged 10–19, dragging the evolution of older adults.

**Figure 4:**
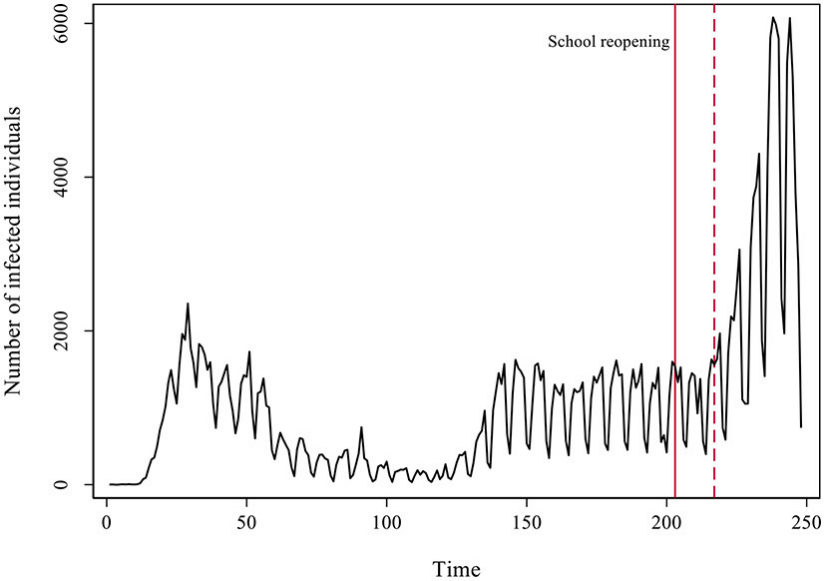
Evolution of the number of COVID-19 cases per day in Catalonia.

In Figure 4 we present the detailed development of the daily number of cases in Catalonia across the two pandemic waves observed. Again, we marked with a red line the moment of the reopening of schools, and with a dotted red line the passing of 14 days of the reopening.

In the first pandemic wave the number of cases observed per day was clearly less than the actual cases due to a lack of testing and plenty underreporting. A remarkable contention of the virus followed the strict lockdown that spanned from mid-March to June the 21st. Infections dropped to a minimum throughout July. During the end of June, cases started raising again but stabilized in a sort of plateau. A plausible explanation for this growth is a concurrent raise in testing during that period, instead of an actual increase in the number of cases (see Supplementary Figure 3).

Besides the growth in testing efforts, the surge in infections that lead to the summer plateau could also be connected to the reopening of bars and restaurants, a share of employees going back to work, and friends and family gatherings. In any case, during that plateau the dynamics was stationary (unit root DF test = −5.187 with a *p*-value = 0.000 for the period). The reopening of schools happened on September 15th and 14 days later a clear exponential growth in the number of cases took place. The co-occurrence in time of two phenomena does not prove causation. The increase in cases could have just temporally coincided with school reopening and be motivated by other factors instead. In any event, school reopening as a cause fulfills one of the rules of causality, that causes must temporally precede effects. Besides that, we already observed a similar pattern across many other ACs. In what follows, we analyze a plausible causal mechanism connecting in-person school reopening with the exponential surge in infections by studying the coevolution of age-groups involved in the process.

**Figure 5:**
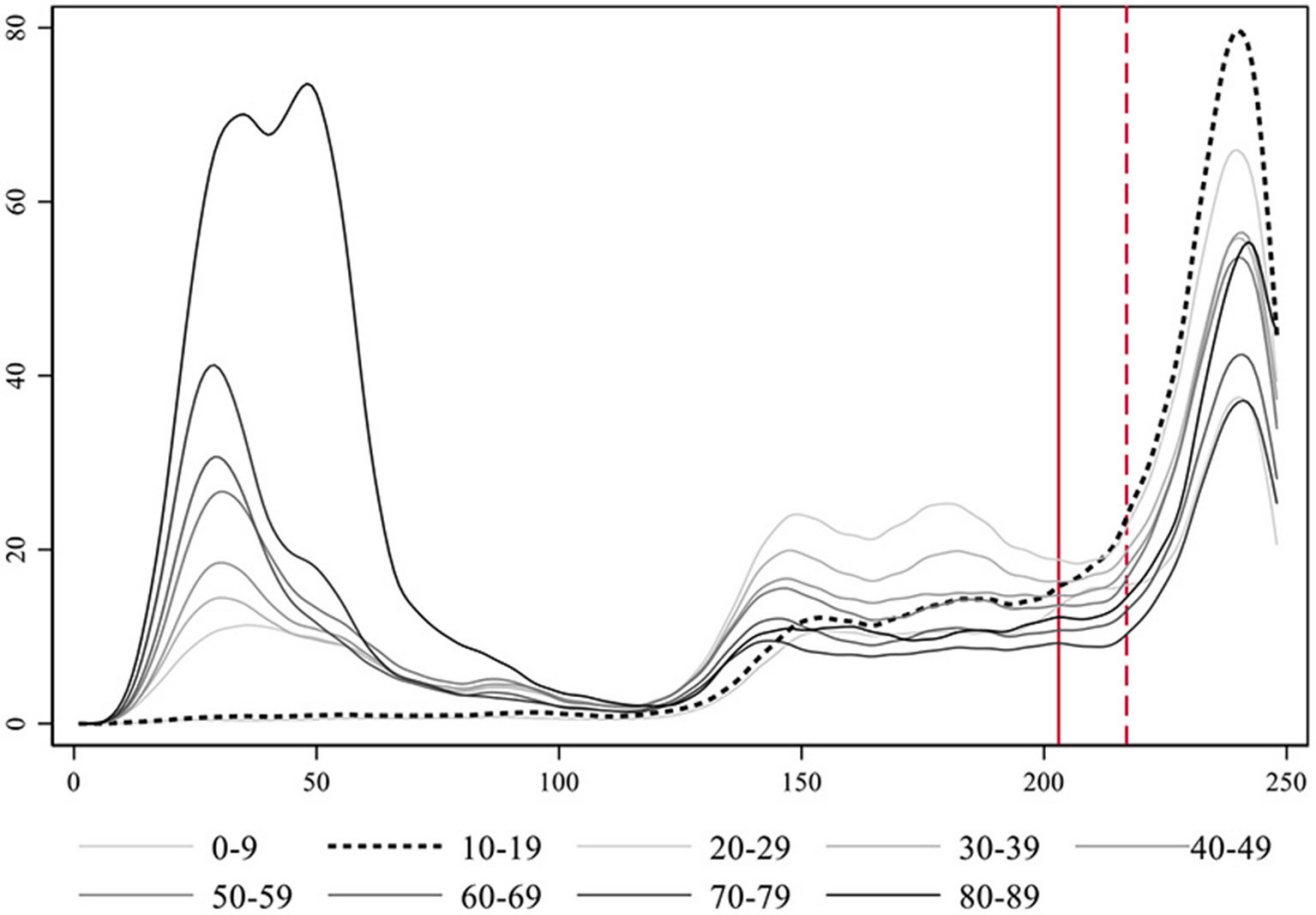
Smoothed estimates using locally weighted regression of the evolution of the rate of COVID-19 cases per day in Catalonia by age-groups. Notice that the age-group 90+ is not represented for clarity purposes, due to its large incidence levels during the first wave.

We implement a smoothing transformation of the time-series data for each age-group with a non-parametric procedure using locally weighted regressions^2^, (see Supplementary Figure 1 to inspect the graphs with the original incidence count data). Figure 5 presents these estimates for all age-groups together, which help to visualize the patterns emerging from the data. The 10–19 age-group is the first experiencing an exponential growth just after school reopening following the plateau phase, and the one with a faster and larger increase in the rate of cases.

**Figure 6:**
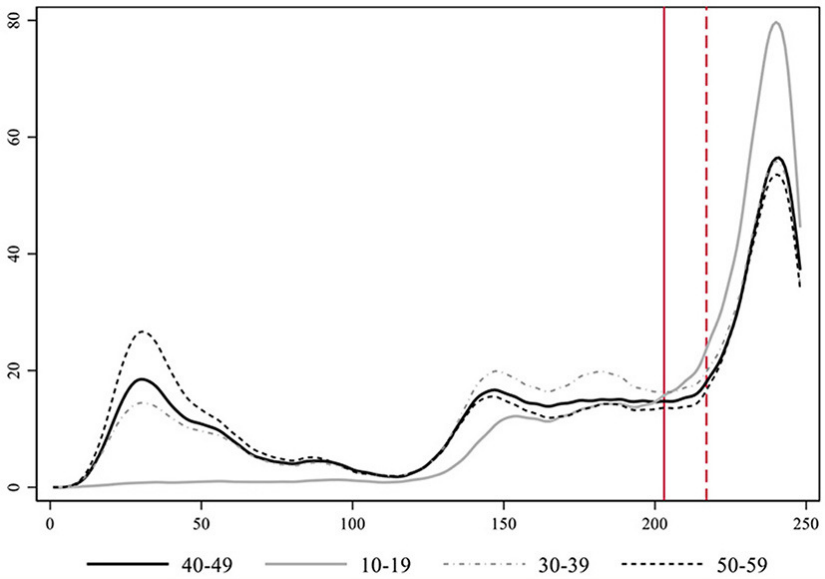
Smoothed estimates using locally weighted regression of the evolution of the number of COVID-19 cases per day in Catalonia by specific age-groups.

We focus on studying people in their forties (40–49 years old) as they are in a stage of the life cycle likely to have school children at home between the ages of 10 and 19^3^. After school reopening, in households where 40-year-olds and their offspring live together, contagion risk would be higher than in other family units. Ever since, not only parents could potentially infect their children but also vice versa. First, we compare the coevolution of youths between 10 and 19 years of age with people in their forties as well as with individuals in their thirties and fifties (Figure 6)^4^. Overall, these three older age-groups are somehow similar in terms of lifestyle and habits. They all loosely belong to the middle-aged category of the human life cycle, clearly differentiated from other life stages such as childhood, youth, or old age. They also portray a similar dynamic.

Our hypothesis regarding the mechanism that connects the evolution of the middle-aged with the 10- to 19-year-old individuals is that they live together in the same households, and the transmission from offspring to adults may have substantially increased due to in-person school reopening. If we compare the development of these two age segments over the period, we see that during the first wave of the pandemic both dynamics were uncorrelated. Middle-aged people got infected, but there were almost no cases (detected) among youths. Besides underreporting and a higher level of asymptomatic cases, schools were closed during the first wave. In the phase after the lockdown, cases among this younger group started to increase, but always remained at a lower level than middle-aged individuals. We consider people in their forties as clearly representative of the middle life stage of the life cycle and the age-group most likely to parent youngster in between 10 and 19 years of age. The plateau phase implied a stationary state for both groups (unit root DF test = −5.323 and −4.963, respectively, with a *p*-value = 0.000). At this stage, the higher level of infections among individuals in their forties could be related to going back to work, and other sort of gatherings. It could well be that, inside the household, contagion from parents to offspring was predominant at that moment. The opening of schools brings a stark increase in youth infections. Cases among younger people (10–19 years old) start rising before the growth among older adults (e.g., 40–49). If we compare a critical range of time, that between September 11th and the 7th of October, infections among youths were increasing faster than among their elders. OLS regressions with deterministic time trends yield a slope of 7.73 in the case of the young age-group and of 5.66 in the older one (full results not shown for simplicity). In fact, the steepest exponential growth of all age-groups takes place among individuals between 10 and 19 years (see Table 2).

**Table 2:** Fitting deterministic linear and exponential time trends to the evolution of the different age-groups over the second pandemic wave in Catalonia.

	***R*** **-squared**		
	**Linear**	**Exponential**	**Ratio**
0–9 years old	0.49	0.62	1.25
10–19 years old	0.45	0.76	1.69
20–29 years old	0.49	0.60	1.23
30–39 years old	0.49	0.59	1.21
40–49 years old	0.47	0.57	1.22
50–59 years old	0.46	0.54	1.18
60–69 years old	0.46	0.55	1.21
70–79 years old	0.44	0.54	1.23
80–89 years old	0.37	0.42	1.13

The smoothing procedure helped us at visually appreciating how the increase in cases among young people aged 10–19 years preceded the subsequent increase in the group aged 40 to 49 and was larger in magnitude. With the aim of testing the role of the 10–19 age-group, after in-person school reopening, to drive the evolution of the pandemic during the second wave through their impact on people of other age-groups, especially middle-aged people, we now perform a set of time series tests using actual incidence rates. As a robustness check, we additionally perform the same tests on a weighted version of the time-series data adjusted for prevalence levels in each age-group.

## Granger causality test

The Granger causality test (51) is a time-series procedure to verify if the evolution of one time series is able to predict another time series. Table 3 shows a group of Granger causality tests to evaluate the effect of the 10–19 age-group series on the 40–49 age-group series. It presents a set of nested OLS regression models with the 40–49 age-group series as the dependent variable and the lagged dependent variable (with up to 10 lags) and the 10–19 age-group variable (also with up to 10 lags) as independent variables. This specification can be expressed using the following equation:


(3) $$\begin{array}{l} y_{t}=\alpha_{0}+(\beta_{1}y_{t-1}+\cdot \cdot \cdot +\beta_{10}y_{t-10})+(\beta_{11}x_{t-1}+\cdot \cdot \cdot \\ +\beta_{20}x_{t-10})+u_{t} \end{array}$$

where the level of infections in the 40–49 age-group *y* at time _*t*_ is a function of a constant α_0_; the lagged dependent variable, in up to 10 consecutive lags (β_1_*y*_*t*−1_ , …, β_10_*y*_*t*−10_); and the lagged independent variable: the level of infections in the 10–19 age-group in up to 10 consecutive lags (β_11_*x*_*t*−1_ , …, β_20_*x*_*t*−10_). The term *u*_*t*_ is the error term of the time-series regression.

**Table 3:** Granger causality test for the 40–49 age-group series as dependent variable.

	**(1)**	**(2)**	**(3)**	**(4)**	**(5)**	**(6)**	**(7)**	**(8)**	**(9)**	**(10)**
	**40–49**	**40–49**	**40–49**	**40–49**	**40–49**	**40–49**	**40–49**	**40–49**	**40–49**	**40–49**
L1.40–49	0.674***	0.579***	0.2	0.255*	0.342***	0.209*	0.152	0.307***	0.344***	0.213*
	(0.115)	(0.142)	(0.134)	(0.141)	(0.129)	(0.122)	(0.113)	(0.11)	(0.11)	(0.115)
L2.40–49		0.221	0.674***	0.717***	0.53***	0.603***	0.431***	0.361***	0.396***	0.34***
		(0.137)	(0.136)	(0.143)	(0.136)	(0.126)	(0.116)	(0.1)	(0.112)	(0.115)
L3.40–49			0.04	−0.029	−0.042	−0.203	−0.131	−0.022	−0.024	0.219*
			(0.123)	(0.147)	(0.139)	(0.131)	(0.12)	(0.104)	(0.098)	(0.115)
L4.40-49				−0.051	0.471***	0.289**	0.148	0.045	0.108	0.133
				(0.124)	(0.134)	(0.129)	(0.119)	(0.103)	(0.098)	(0.096)
L5.40–49					−0.514***	−0.142	−0.331***	−0.163	−0.223**	−0.215**
					(0.114)	(0.127)	(0.118)	(0.103)	(0.098)	(0.096)
L6.40–49						0.051	−0.02	0.173*	0.221**	0.217**
						(0.118)	(0.117)	(0.103)	(0.098)	(0.096)
L7.40–49							0.664***	0.513***	0.632***	0.645***
							(0.108)	(0.1)	(0.098)	(0.096)
L8.40–49								−0.349***	−0.448***	−0.274**
								(0.106)	(0.113)	(0.121)
L9.40–49									−0.16	−0.08
									(0.106)	(0.116)
L10.40–49										−0.407***
										(0.105)
L1.10–19	0.089	0.11	0.408***	0.397***	0.34***	0.348***	0.292***	0.314***	0.102	0.185*
	(0.075)	(0.111)	(0.104)	(0.105)	(0.096)	(0.095)	(0.093)	(0.086)	(0.096)	(0.096)
L2.10–19		−0.096	−0.822***	−0.876***	−0.858***	−0.825***	−0.58***	−0.435***	−0.236**	−0.256**
		(0.102)	(0.12)	(0.127)	(0.116)	(0.108)	(0.103)	(0.091)	(0.104)	(0.114)
L3.10–19			0.413***	0.525***	0.638***	0.563***	0.411***	0.279***	0.281***	0.183*
			(0.089)	(0.131)	(0.127)	(0.118)	(0.109)	(0.095)	(0.09)	(0.104)
L4.10–19				−0.038	−0.625***	−0.314**	−0.222**	−0.161*	−0.186**	−0.201**
				(0.094)	(0.123)	(0.123)	(0.112)	(0.096)	(0.091)	(0.089)
L5.10–19					0.603***	0.036	0.207*	0.199**	0.214**	0.198**
					(0.086)	(0.12)	(0.113)	(0.097)	(0.092)	(0.09)
L6.10–19						0.295***	0.157	−0.113	−0.083	−0.075
						(0.092)	(0.109)	(0.098)	(0.093)	(0.09)
L7.10–19							−0.224**	0.166*	0.021	0.021
							(0.091)	(0.095)	(0.093)	(0.091)
L8.10–19								−0.186**	0.166	0.048
								(0.085)	(0.113)	(0.116)
L9.10–19									−0.213**	−0.192
									(0.094)	(0.122)
L10.10–19										0.189**
										(0.094)
Constant	3.897***	3.037***	1.546	1.734	2.126**	1.903**	1.26	1.416*	1.558**	1.921***
	(1.057)	(1.169)	(1.051)	(1.082)	(1.009)	(0.949)	(0.871)	(0.754)	(0.721)	(0.713)
Observations	259	258	257	256	255	254	253	252	251	250
*R*–squared	0.648	0.652	0.743	0.744	0.79	0.822	0.856	0.895	0.907	0.913
**Granger test**										
*F*	1.41	0.56	16.50	12.13	21.93	15.33	6.89	4.94	3.67	3.47
Sig.	0.236	0.572	0.000	0.000	0.000	0.000	0.000	0.000	0.000	0.000

Standard errors are in parentheses.

^***^p < 0.01,

^**^p < 0.05,

^*^p < 0.1.

We are interested in the *F*-statistic of the models that will eventually allow us to reject the null hypothesis. We reject the null in eight of the 10 models. Only with lags one and two the *F*-statistic is below the critical threshold. This has a substantive meaning: it takes longer than one or two single lags for the dynamics of the 10–19 age-group series to influence the 40–49 series. In the remaining models with more lags, the *p*-value associated to the *F*-statistic is always under 0.05 (*p* < 0.000) indicating that we can reject the null hypothesis that all coefficients of lag of the independent variable (10–19 age group series) are equal to 0. Therefore, we can state that the 10–19 age-group Granger causes the 40–49 age-group series.

Instead of this stream of causality from children to adults, could the level of parental infections be driving the level of infections of their offspring? To test it, we reverted the former Granger causality analysis so that the 10–19 age-group series is now the dependent variable (*y*_*t*_) and the 40–49 series the independent variable (*x*_*t*−*n*_). This would allow us checking whether there is a sort of reverse process by which the 40-year-olds are those who cause youths to get infected. As shown in Table 4, there is also evidence of this line of causation, but it is substantially weaker. In only three of the 10 models, we observe a Granger causal process. In any case, a bidirectional association among both series is consistent with the notion of a feedback relationship due to cohabitation of these age-groups in the same family units within households.

**Table 4:** Granger causality test for the 10–19 age–group series as dependent variable.

	**(1)**	**(2)**	**(3)**	**(4)**	**(5)**	**(6)**	**(7)**	**(8)**	**(9)**	**(10)**
	**10–19**	**10–19**	**10–19**	**10–19**	**10–19**	**10–19**	**10–19**	**10–19**	**10–19**	**10–19**
L1.10–19	0.726***	0.808***	1.119***	1.097***	0.992***	0.943***	0.771***	0.943***	0.782***	0.845***
	(0.096)	(0.143)	(0.14)	(0.141)	(0.124)	(0.115)	(0.118)	(0.099)	(0.115)	(0.117)
L2.10–19		−0.041	−0.878***	−0.962***	−0.944***	−0.862***	−0.631***	−0.374***	−0.203	−0.178
		(0.132)	(0.16)	(0.17)	(0.149)	(0.131)	(0.131)	(0.104)	(0.125)	(0.139)
L3.10–19			0.528***	0.624***	0.691***	0.541***	0.41***	0.212*	0.225**	0.1
			(0.119)	(0.176)	(0.163)	(0.143)	(0.138)	(0.108)	(0.108)	(0.127)
L4.10–19				0.045	−0.727***	−0.231	−0.178	−0.086	−0.104	−0.125
				(0.125)	(0.159)	(0.149)	(0.142)	(0.11)	(0.109)	(0.109)
L5.10–19					0.966***	−0.022	0.192	0.195*	0.208*	0.201*
					(0.111)	(0.145)	(0.143)	(0.111)	(0.11)	(0.11)
L6.10–19						0.636***	0.291**	−0.111	−0.091	−0.091
						(0.112)	(0.138)	(0.112)	(0.111)	(0.11)
L7.10–19							0.12	0.847***	0.765***	0.764***
							(0.115)	(0.109)	(0.112)	(0.111)
L8.10–19								−0.636***	−0.375***	−0.458***
								(0.098)	(0.136)	(0.142)
L9.10–19									−0.223**	−0.272*
									(0.112)	(0.149)
L10.10–19										0.23**
										(0.115)
L1.40–49	0.225	0.205	−0.19	−0.117	0.027	−0.153	−0.126	−0.108	−0.044	−0.134
	(0.147)	(0.183)	(0.18)	(0.188)	(0.166)	(0.148)	(0.143)	(0.125)	(0.132)	(0.14)
L2.40–49		−0.061	0.488***	0.573***	0.368**	0.498***	0.322**	0.199*	0.149	0.072
		(0.176)	(0.182)	(0.191)	(0.175)	(0.152)	(0.148)	(0.115)	(0.134)	(0.141)
L3.40–49			−0.056	−0.08	0.002	−0.253	−0.146	0.051	0.038	0.263*
			(0.165)	(0.196)	(0.18)	(0.158)	(0.151)	(0.119)	(0.118)	(0.14)
L4.40–49				−0.193	0.568***	0.317**	0.145	−0.038	0.009	0.035
				(0.166)	(0.173)	(0.155)	(0.151)	(0.118)	(0.118)	(0.117)
L5.40–49					−1.001***	−0.308**	−0.451***	−0.176	−0.22*	−0.227*
					(0.147)	(0.154)	(0.149)	(0.118)	(0.118)	(0.118)
L6.40–49						−0.141	−0.032	0.193	0.225*	0.234**
						(0.142)	(0.148)	(0.118)	(0.118)	(0.118)
L7.40–49							0.309**	−0.063	−0.012	−0.01
							(0.137)	(0.115)	(0.118)	(0.117)
L8.40–49								−0.082	−0.197	−0.068
								(0.122)	(0.136)	(0.147)
L9.40–49									0.027	0.137
									(0.127)	(0.142)
L10.40–49										−0.377***
										(0.128)
Constant	0.564	1.252	−0.273	0.127	1.131	0.987	0.635	0.733	0.768	1.086
	(1.354)	(1.503)	(1.407)	(1.447)	(1.301)	(1.147)	(1.104)	(0.864)	(0.865)	(0.869)
Observations	259	258	257	256	255	254	253	252	251	250
*R*–squared	0.753	0.754	0.804	0.806	0.852	0.89	0.902	0.942	0.944	0.946
**Granger tests**										
*F*	2.34	0.67	2.59	2.41	10.89	4.04	2.91	1.10	1.20	1.93
Sig.	0.127	0.510	0.053	0.050	0.000	0.001	0.001	0.366	0.295	0.043

Standard errors are in parentheses.

^***^p < 0.01,

^**^p < 0.05,

^*^p < 0.1.

## Chow test

In addition, we may want to verify when this relationship between the two time-series appears. We perform a test to check whether the opening of schools, as an external shock, implies a key disruption in the series under study here (Table 5). The Chow test is calculated after an OLS regression with the lagged dependent variable and the lagged independent variable as regressors together with the interaction of school reopening with both age-group series. The equation can be portrayed as follows:


(4) $$\begin{array}{l} y_{t}=\alpha_{0}+\beta_{1}y_{t-1}+\beta_{2}x_{t-1}+\beta_{3}z+\beta_{4}\left(y_{t-1}\cdot z\right)+\beta_{5}\left(x_{t-1}\cdot z\right)+u_{t} \end{array}$$

where the level of infections in the 40–49 age-group *y* at time _*t*_ is a function of a constant α_0_, the lagged dependent variable β_1_*y*_*t*−1_), the lagged independent variable (the level of infection of the 10–19 age-group expressed by β_2_*x*_*t*−1_), a dummy variable representing school reopening β_3_*z*, the interaction of school reopening with the lagged dependent variable β_4_(*y*_*t*−1_·*z*) and the lagged independent variable β_5_(*x*_*t*−1_·*z*). The term *u*_*t*_ is the error term of the time-series regression.

**Table 5:** Chow test of school reopening.

	**Coef**.		**SE**
Constant	2.71*		1.196
40–49 years old			
Lag 1	0.721**		0.151
10–19 years old			
Lag 1	0.028		0.151
School opening	10.42**		2.732
School * 40–49	0.104		0.261
School * 10–19	−0.161		0.213
Observations		259	
*R*-squared		0.676	
**Chow test**			
*F*		7.230	
*P*-value		0.000	

^**^p < 0.01,

^*^p < 0.05.

The null hypothesis for the Chow test means no break. If the *p*-value is < 0.05, we can reject the null in favor of the alternative that there is a break. Our results indicate that the null hypothesis can be rejected, and we can conclude that school reopening caused a break in the regression coefficients.

## Weighting by prevalence as a robustness check

The use of actual incidence records involves assuming that measurement error does not substantially distort our inferences. The proportion of asymptomatic cases is a key aspect to understand the pattern of the SARS-CoV-2 spread. Previous research (52) establishes that almost 60% of infected people report no symptomatology during an early stage of the disease, although symptoms can appear later as a result of being tested in the presymptomatic phase (53). This serves as a basis to discuss regarding the proportionality of the diagnostic effort done in all the age-groups, and if this can affect the analysis. Some studies suggested that the age range from 0 to 20 is highly asymptomatic (54). Moreover, other analyses seem to show that the prevalence on children is higher than previously thought (55), being prevalence a good estimator for capturing the true incidence on the population. Therefore, raw incidence data certainly contains statistical biases due to non-random factors such as the degree of asymptomatic individuals, which vary by age-group, or differences in diagnosis efforts on each age-segment of the population. In contrast to official incidence records, prevalence studies are implemented using random sampling, which allows obtaining more representative and realistic incidence estimates by age-groups. When data does not come from a random sample, as in the official records of infected individuals, it is susceptible of containing systematic error from the self-selection of symptomatic infected individuals that correlates with aging, or to over represent certain population segments for whom public diagnosis efforts are higher, such as younger individuals, but using less representative sampling procedures. The number of tests done for the age group from 10 to 19 is huge as compared to those performed on other age groups (see Supplementary Figure 3). However, this does not imply an improvement in detection, since it depends on the method used to perform the testing, and on whether the samples are correctly selected. As an example, the tests performed in a classroom typically composed of 25 students due to the detection of a positive index case will result in largely negative tests results. This is because only about 8% of infective individuals are responsible for 60% of the cases (56), and highly asymptomatic individuals are less infectious (57).

As a robustness test of our main analysis, we use data from large scale prevalence studies in Spain (42–45) to weight the actual incidence records and try compensating for the aforementioned biases. This robustness test implies weighting each age-group's time-series by their specific percentual level of detection before reanalyzing the data (see the explanation of the calculation procedure on the Supplementary material).

**Figure 7:**
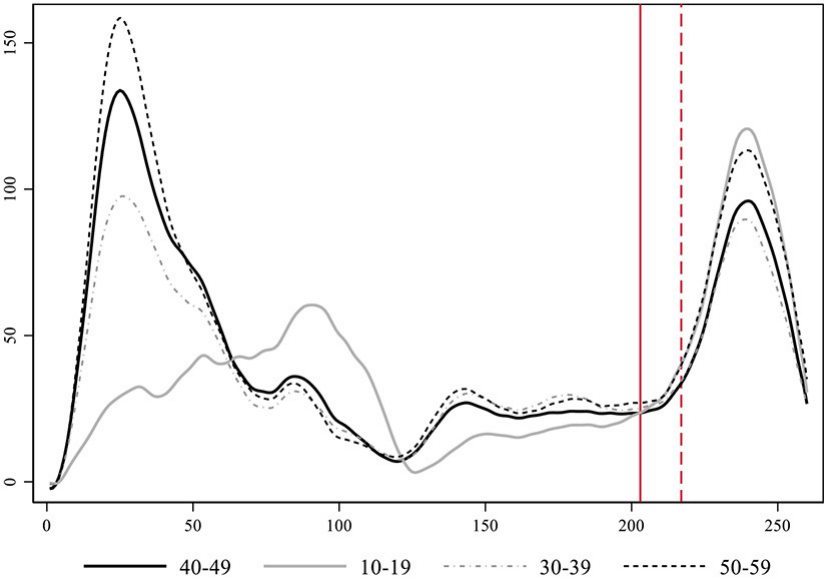
Evolution of the rate of COVID-19 cases per day in Catalonia by age-group with data corrected by the level of prevalence.

In Figure 7 we present the smoothed estimates of the incidence rates weighted by prevalence. Again, we can clearly identify how the 10–19 series grows exponentially faster and more intensely than the middle-aged series just after school reopening during the second wave. The increase among the 50-year-old individuals becomes now the second in importance. Furthermore, weighted data allows appreciating a more realistic estimate of the true overall magnitude of the first wave, which was far wider than the second. In any case, incidence among youths (10–19) during the first wave was rather low coinciding with a period when in-person school was closed.

The 10–19 age-group moves from being the second least infected group during the first wave (just after children between 0 and 9 years of age as shown in Supplementary Figure 5) to be leading the levels of contagion during the second wave, both in terms of the timing an intensity of its growth. The key exogenous contextual element that varies between these two waves and may be responsible for this difference is in-person schooling. Contagion among youths related to in-person classes does not necessarily translate into a life-threatening health risk for this age-group, however, it increases the danger in aggregate terms for the transmission to individuals of older age-groups across society at large, and especially within the multigenerational households where these young people live. Figure 7 further allows appreciating an overlooked increased in contagion of youths taking place after the first wave, when containment measures were slightly relaxed. This growth finally went down during the summer months. If we rerun the Granger causality tests specified with the 40–49 series as determined by the 10–19 and up to 10 lags, we obtain evidence of Granger causality processes in three of its lags (see Supplementary Table 3). All in all, the replication of the analysis on the weighted time-series increases our confidence in the validity of our main results.

## Discussion

In-person school reopening taking place at different dates during September 2020 precedes and correlates with a posterior growth in contagion in almost all Spanish regions. The time-series analysis of Catalan age-groups indicates that contagion among young individuals aged 10–19 after school-reopening grows earlier and faster than the rest, Granger causing the evolution of other age-groups. The lack of public awareness of this phenomenon might be due to a collective cognitive confusion regarding the actual role of school reopening. Different studies at the individual level show that children become less infected and are less infectious than older individuals (58). From this fact many conclude that it was rather safe to keep schools opened. This inference could actually be a sort of fallacy. Even if children are less infectious and get less infected (some estimates say by half), it has been proven they are infectious and infect. Moreover, even a smaller proportion of infected individuals can imply a large number of actual cases when the target population is very large: the whole school children population. As a result, the aggregate role of opening schools for the expansion of the pandemic can be collectively underestimated.

Like almost all the statistical tests, there is strong evidence for the correlation of the different effects presented here, but the causation if hidden. Granger causality is a statistical hypothesis test for determining whether one time series can forecast another one. Notice that it is only capable of testing the temporal relation between the two time-series, since the true causation is a complex philosophical issue, here we can only assess if one time-series forecasts another time-series.

To test the robustness of our findings we implemented a weighting procedure based on prevalence studies to estimate the actual percent of detection. This allows us to generate a new time-series that represents the real cases. Reproducing our analysis with this corrected data yield equivalent results, enforcing our confidence in the findings.

Other studies have also analyzed the impact of school closures together with other non-pharmacological interventions. These studies employed large datasets that included multiple countries and various non-pharmacological interventions, and in all of them it was observed that the closure of schools provided a reduction in the *R*_t_ (6–8, 59). School reopening seems to have an impact on the *R*_t_ when this non-pharmaceutical intervention is lifted and applied, and coherently with the mortality (21), as is described on (60).

Furthermore, another study (61), using a methodology similar to that employed in our research, observed that parental exposure to open schools is associated with a somewhat higher rate of PCR-confirmed SARS-CoV-2 infection OR 1.17; CI 95% 1.03–1.32. It was also higher among teachers, PCR-confirmed SARS-CoV-2 infection OR 2.01; CI 95% 1.52–2.67.

In addition, a different research (62) robustly estimated that the closure of schools, like other interventions to reduce contacts in large groups, is one of the most effective interventions to contain the spread of COVID-19 by reducing the daily incidence.

While previous research has identified the overall impact of different non-pharmacological interventions in the reduction of SAR-CoV-2 spread, our study focuses more in depth on one of those interventions (school closure/reopening), in a specific context (Spain and Catalonia), at a particular moment in time (the second wave) and using an interrupted time-series approach. Our method can be easily reproduced in other countries to eventually find comparable patterns.

From our analysis we can contemplate the possibility that school reopening may generate a retro-feedback with parents' return to work and social activity, leading to an exponential growth, as observed in Catalonia and other Spanish ACs during September and October of 2020.

Despite its cost, online or hybrid schooling could have been a cost-effective option considering the potential role of schools as drivers of the virus in the community. The spread of the virus may imply higher expenses when medical, economic, and social costs of closing economic activities due to the arrival of a new viral wave are contemplated altogether. This understanding could help policy makers to find suitable solutions to limit the spread of the virus in the community such as using tele-education while keeping onsite schools for parents that need it, improving the ventilation of classes with HEPA filters, or reducing the ratios for onsite school.

Posterior virus variants, such as the B.1.1.7 detected in the UK, seem to increase the transmission rate among children. If this is confirmed, new analysis should be performed to assess how it will amplify the transmission rate in the community. The estimated effects of school reopening would constitute a downward estimation of the real impact in a context where new variants are widespread.

All in all, the findings presented here are consequential not only for the particular case of study, but more generally. Heated debates about the adequacy and safety of in-person school reopening have been held around the world. Different considerations regarding its costs and benefits have been casted, however, the full implications of its costs might not been weighted accurately enough. We believe our findings constitute a contribution in this direction.

## Data availability statement

Publicly available datasets were analyzed in this study. This data can be found at: Generalitat de Catalunya, Registre de casos de COVID-19 realitzats a Catalunya. Segregació per sexe i edat. Dades Obertes (2020; February 21, 2021) Tyler, Socrata Open Data Server. Socrata (2020; June 2, 2020). CNE, Incidencias acumuladas e indicadores de transmisibilidad (2020; February 21, 2021).

## Author contributions

RT: statistical modeling and analysis and scientific writing. PF: data preparation and literature review. JG-A: review and additional help. All authors contributed to the article and approved the submitted version.

## Funding

This research was partially funded by the CCD of Universitat Politècnica de Catalunya, grant 2020–L015.

## Conflict of interest

The authors declare that the research was conducted in the absence of any commercial or financial relationships that could be construed as a potential conflict of interest.

## Publisher's note

All claims expressed in this article are solely those of the authors and do not necessarily represent those of their affiliated organizations, or those of the publisher, the editors and the reviewers. Any product that may be evaluated in this article, or claim that may be made by its manufacturer, is not guaranteed or endorsed by the publisher.

## Footnotes

^1^The estimates of an equivalent fixed effects model adjusting for time-invariant unobserved heterogeneity portrays the exact same estimates as the random effects model (see Supplementary Table 2).

^2^Using the lowess command in Stata.

^3^According to official statistics (INE), in Catalonia the average age for a woman to have a child during the period from 2000 to 2019 was 31.13 years, the highest in Europe (48).

^4^Analyses including the other middle-aged groups are included in the Supplementary Figure 1.
