# Supplementary material for: In-person school reopening and the spread of SARS-CoV-2 during the second wave in Spain
Source: Front Public Health. 2022 Oct 13;10:990277. doi: 10.3389/fpubh.2022.990277 (PMC9608566; doi:10.3389/fpubh.2022.990277)
Supplement: Supplementary file 1 [file Data_Sheet_1.docx]

**Supplementary Information for**

In-Person School Reopening and the Spread of SARS-CoV-2 During the Second Wave in Spain

Supplementary Text

**Percent of detection calculus.** Our purpose is to use the prevalence data to estimate the percentage of detection. We chose to analyze this percent of detection in two time periods: Round III (which includes all previous rounds); and the increase the prevalence of Round IV with respect to III, which also includes the eventual impact of school opening. The justification to divide the analysis between Round I to III and the increase of Round IV is due to the prevalence for our interest groups for the Rounds I, II and III remains almost the same, see Fig S2, showing that the cases do not increases on this period (the summer plateau).

The prevalence data is provided by age-groups for the total Spanish ACs and also for each AC. Notice that from this prevalence one can calculate the percent of detection for each age group, since the detected cases is a known variable, see Table S5, and we can calculate form the prevalence an estimation of the real cases. As example, for Catalonia we have a percent of detection of about 60% at the end of Round IV, see Table S6. We assume that the distribution of prevalence levels by age-group is similar across the different ACs. Therefore, to estimate the prevalence by age-group, for the case of Catalonia, we use the Catalonia prevalence, Table S6, and we weight it by the prevalence by age-groups of the total of Spanish the ACs of Table S5. We can calculate the percent of detection for Catalonia for the different age ranges with the weighted prevalence for the Round I to III, Table S7, and for the increase of the prevalence of Round IV, Table S8.

We divided the time series in two periods, one for the period that encompasses the data until the end of the Round IIII of the prevalence analysis, Table S7, and another until the end of the Round IV of the analysis, Table S8. At this point we can generate a new dataset containing an estimation of the daily real cases, using the detected cases corrected by the percent of detection calculated from the prevalence.

Fig. S1. Evolution of the number of COVID19 cases per day in Catalonia among individuals between 40 and 49 years of age and individuals between 10 and 19.


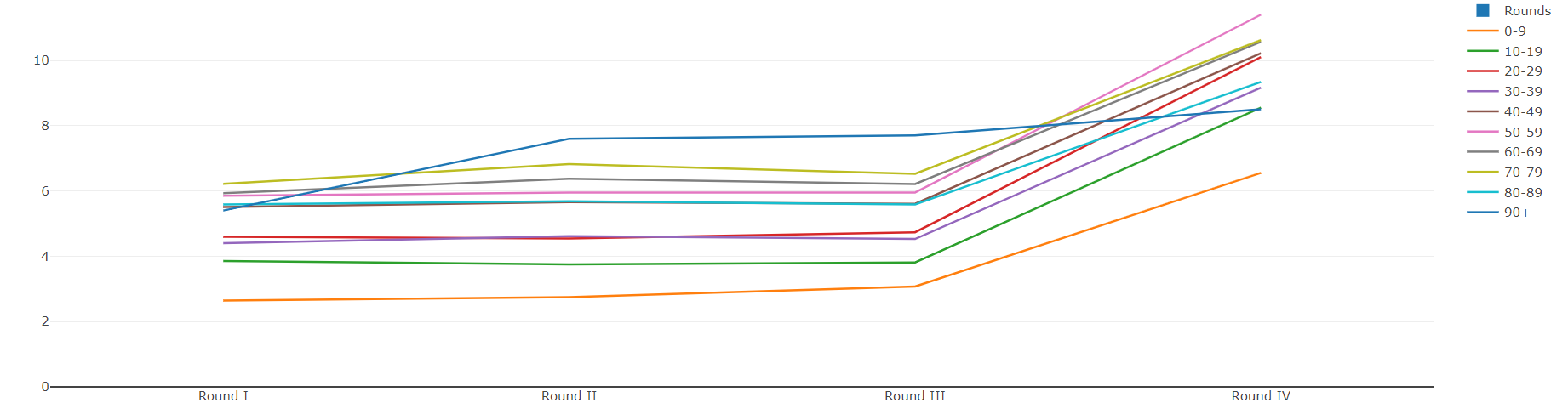


Fig S2. Prevalence by ages for the different rounds. Notice that the prevalence remains almost flat from Round I to III, only changing clearly in Round IV that includes the impact of second wave of the pandemic in Spain.


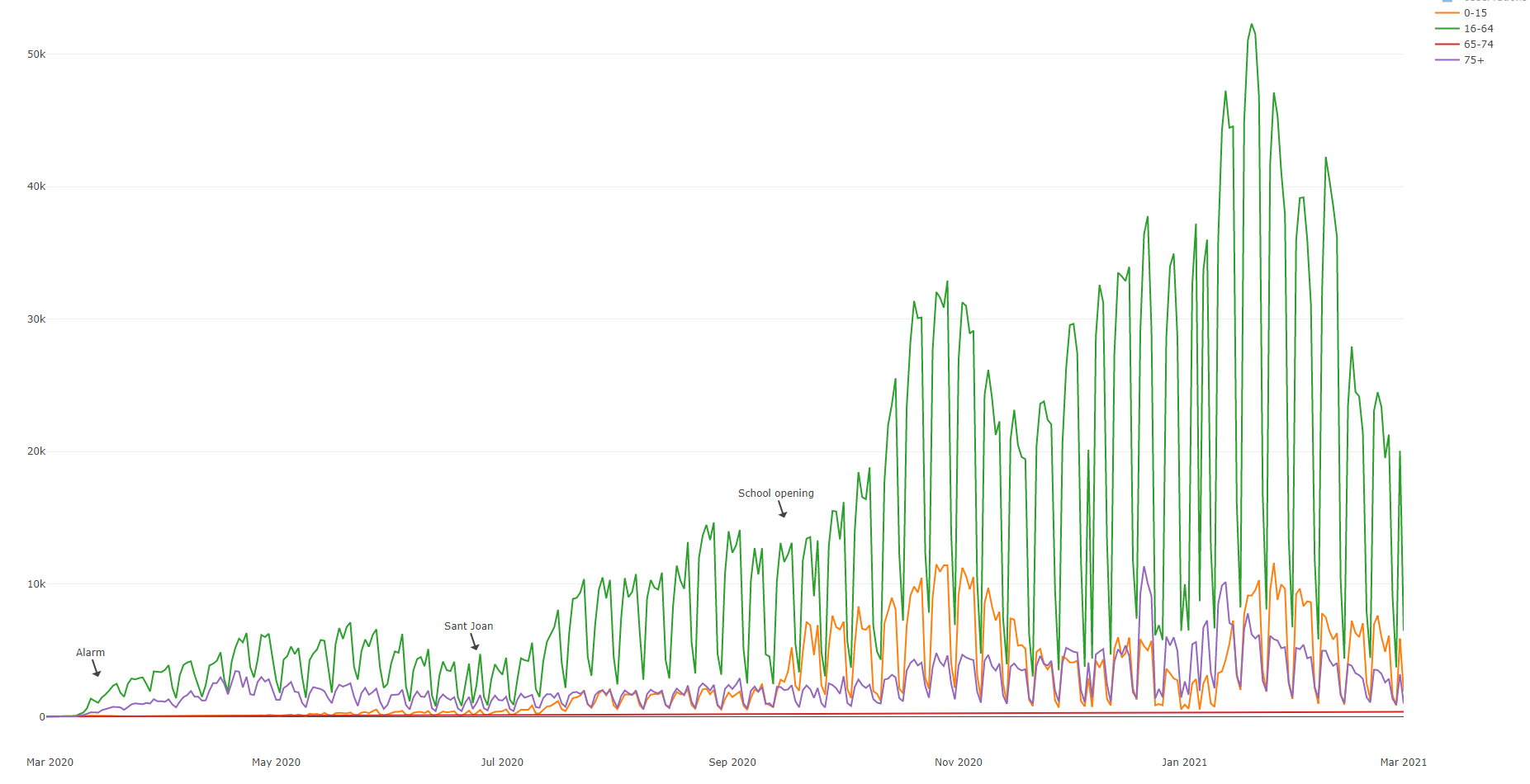


Fig S3. Testing done for age ranges. Notice the increase of the number of tests done for all age groups but specifically for 0-15 age group when schools reopened.

**

Fig S4. Evolution of the rate of COVID19 cases per day in Catalonia by age-group with data corrected by the level of prevalence.

# Table S1. Poisson generalized linear regression models adjusted for overdispersion to test the effect of school reopening on each AC. Estimates being Incidence-Rate Ratios

|  | **AN** | **AR** | **AS** | **CB** | **CE** | **CL** | **CM** | **CN** | **CT** | **EX** |
| --- | --- | --- | --- | --- | --- | --- | --- | --- | --- | --- |
| Time | 1.016*** | 1.016*** | 1.007*** | 1.011*** | 1.016*** | 1.007*** | 1.004*** | 1.016*** | 1.007*** | 1.009*** |
|  | (.002) | (.001) | (.002) | (.002) | (.001) | (.001) | (0) | (.001) | (.001) | (.001) |
| School reopening | 1.654*** | .735** | 4.680*** | 1.160 | 2.952*** | 2.662*** | 2.790*** | .396*** | 1.883*** | 3.174*** |
|  | (.284) | (.090) | (1.051) | (.245) | (.611) | (.483) | (.559) | (.069) | (.292) | (.597) |
| Constant | 12.078*** | 9.361*** | 5.470*** | 4.156*** | .120*** | 58.58*** | 70.57*** | 2.781*** | 175.6*** | 7.748*** |
|  | (3.816) | (1.190) | (1.514) | (1.189) | (.043) | (12.27) | (13.96) | (.811) | (31.29) | (1.846) |
| Observations | 316 | 316 | 316 | 316 | 316 | 316 | 316 | 316 | 316 | 316 |
| Deviance | 77,618.6 | 26,888.9 | 12,947.9 | 11,283.2 | 1,173.3 | 71,353.3 | 58,166.5 | 15,319.6 | 161,985.0 | 13,909.7 |
| Pearson | 122,933.7 | 30,671.1 | 16,632.0 | 16,713.6 | 1,629.3 | 89,800.1 | 77,846.9 | 20,531.4 | 196,400.9 | 18,894.8 |
| AIC | 251.48 | 90.53 | 44.49 | 39.39 | 5.68 | 231.51 | 189.56 | 52.60 | 519.73 | 48.29 |
| BIC | 75,817.06 | 25,087.31 | 11,146.31 | 9,481.65 | -628.24 | 69,551.73 | 56,364.91 | 13,518.06 | 160,183.5 | 12,108.13 |

|  | **GA** | **IB** | **MC** | **MD** | **ML** | **NC** | **PV** | **RI** | **VC** |  |
| --- | --- | --- | --- | --- | --- | --- | --- | --- | --- | --- |
| Time | 1.007*** | 1.018*** | 1.020*** | 1.006*** | 1.016*** | 1.007*** | 1.011*** | 1.004*** | 1.012*** |  |
|  | (.001) | (.001) | (.001) | (.001) | (.001) | (.001) | (.001) | (.001) | (.001) |  |
| School reopening | 2.008*** | .367*** | 1.090 | 1.663** | 2.724*** | 3.159*** | 1.088*** | 2.839*** | 1.176 |  |
|  | (.388) | (.059) | (.152) | (.330) | (.497) | (.523) | (.183) | (.625) | (.206) |  |
| Constant | 25.661*** | 2.098*** | 1.568 | 278.6*** | .161*** | 17.944*** | 25.511*** | 15.929*** | 22.809*** |  |
|  | (5.774) | (.594) | (0.439) | (57.75) | (.051) | (3.301) | (5.854) | (3.304) | (5.501) |  |
| Observations | 316 | 316 | 316 | 316 | 316 | 316 | 316 | 316 | 316 |  |
| Deviance | 34,920.6 | 12,677.5 | 15,583.9 | 303,959.1 | 1,743.3 | 17,956.6 | 51,556.3 | 14,034.2 | 48,700.8 |  |
| Pearson | 46,296.5 | 17,814.1 | 19,954.5 | 374,986.6 | 1,868.6 | 21,506.6 | 68,377.0 | 18,192.3 | 68,811.1 |  |
| AIC | 115.33 | 47.57 | 53.66 | 969.15 | 7.60 | 61.64 | 168.75 | 48.23 | 159.71 |  |
| BIC | 33,119.03 | 11,875.93 | 13,782.32 | 302,157.6 | -58.27 | 16,155.05 | 49,754.77 | 12,232.64 | 46,899.2 |  |
| *Observed information matrix standard errors are in parentheses.* | | | |  |  |  |  |  |  |  |
| **** p<.01, ** p<.05, * p<.1* | | |  |  |  |  |  |  |  |  |

**Table S2**. Fixed and random effects regressions with entity-specific intercepts (ACs).

|  |  |  |
| --- | --- | --- |
|  | **Fixed effects** | **Random effects** |
| School reopening | 1.760*** | 1.760*** |
|  | (0.005) | (0.005) |
| Time | 1.009*** | 1.009*** |
|  | (0.000) | (0.000) |
| Constant |  | 36.167*** |
|  |  | -8.699 |
| Ln Alpha |  | 0.15 |
| Alpha |  | 1.162 |
| Log likelihood | -538,678.63 | -538,910.76 |
| Observations | 5,966 | 5,966 |
| *The estimates of Poisson models are incidence rate ratios.* | | |
| *Standard errors are in parentheses* | | |
| **** p<.01, ** p<.05, * p<.1* | | |

# Table S3. Poisson generalized linear regression models adjusted for overdispersion to test the effect of pre-secondary school reopening (a) as compared to (b) secondary school reopening on each AC. Estimates being Incidence-Rate Ratios.

|  | **Andalusia** | | **Aragon** | | **Asturias** | | **Cantabria** | |
| --- | --- | --- | --- | --- | --- | --- | --- | --- |
|  | (a) | (b) | (a) | (b) | (a) | (b) | (a) | (b) |
| Time | 1.016*** | 1.017*** | 1.016*** | 1.016*** | 1.008*** | 1.007*** | 1.010*** | 1.011*** |
|  | (.002) | (.002) | (.001) | (.001) | (.002) | (.002) | (.002) | (.001) |
| School reopening | 1.687*** | 1.654*** | 0.706*** | 0.735** | 4.593*** | 2.662*** | 1.353 | 1.160 |
|  | (.306) | (.284) | (.089) | (.090) | (1.087) | (1.051) | (.291) | (.245) |
|  |  |  |  |  |  |  |  |  |
| Constant | 11.959*** | 12.078*** | 9.038*** | 9.361*** | 5.204*** | 5.470*** | 4.666*** | 4.156*** |
|  | (3.816) | (3.816) | (1.850) | (1.899) | (1.494) | (1.514) | (1.293) | (1.189) |
| Observations | 316 | 316 | 316 | 316 | 316 | 316 | 316 | 316 |
| AIC | 251.720 | 251.477 | 90.139 | 90.525 | 45.269 | 55.487 | 39.146 | 39.391 |
| BIC | 75893.61 | 75817.76 | 24965.22 | 25087.31 | 11393.44 | 11146.31 | 9404.44 | 9481.65 |

|  | **Castile and León** | | **Canary Island** | | **Galicia** | | **Region of Murcia** | |
| --- | --- | --- | --- | --- | --- | --- | --- | --- |
|  | (a) | (b) | (a) | (b) | (a) | (b) | (a) | (b) |
| Time | 1.006*** | 1.007*** | 1.016*** | 1.016*** | 1.007*** | 1.007*** | 1.019*** | 1.020*** |
|  | (.001) | (.001) | (.001) | (.001) | (.001) | (.001) | (.001) | (.001) |
| School reopening | 3.109*** | 2.662*** | .405 | .396*** | 2.073*** | 2.008*** | 1.330** | 1.009 |
|  | (.590) | (.483) | (.071) | (.069) | (.420) | (0.388) | (.188) | (.152) |
|  |  |  |  |  |  |  |  |  |
| Constant | 63.731*** | 58.581*** | 2.836 | 2.781*** | 26.109*** | 25.661*** | 1.980*** | 1.568 |
|  | (13.108) | (12.273) | (.825) | (.810) | (5.949) | (5.774) | (.517) | (.439) |
| Observations | 316 | 316 | 316 | 316 | 316 | 316 | 316 | 316 |
| AIC | 225.195 | 231.509 | 52.939 | 52.599 | 115.275 | 115.332 | 52.962 | 53.658 |
| BIC | 67556.54 | 69551.73 | 13625.79 | 13518.06 | 33101.17 | 33119.03 | 13562.37 | 13782.32 |
| *Observed information matrix standard errors are in parentheses.*  **** p<.01, ** p<.05, * p<.1*   1. *Preschool and primary education* 2. *Secondary education* | | | |  |  |  |  |  |

# Table S4. Granger causality test for the 40-49 age-group series as dependent variable with data weighted by prevalence.

|  | **(1)** | **(2)** | **(3)** | **(4)** | **(5)** | **(6)** | **(7)** | **(8)** | **(9)** | **(10)** |  |
| --- | --- | --- | --- | --- | --- | --- | --- | --- | --- | --- | --- |
|  | 40-49 | 40-49 | 40-49 | 40-49 | 40-49 | 40-49 | 40-49 | 40-49 | 40-49 | 40-49 |  |
| L.40-49 | .844*** | .674*** | .601*** | .578*** | .58*** | .563*** | .476*** | .745*** | .584*** | .488*** |  |
|  | (.044) | (.076) | (.076) | (.08) | (.08) | (.076) | (.068) | (.063) | (.07) | (.071) |  |
| L2.40-49 |  | .207*** | .045 | .043 | .03 | .024 | .054 | .008 | .203*** | .114 |  |
|  |  | (.076) | (.086) | (.087) | (.089) | (.084) | (.075) | (.062) | (.076) | (.077) |  |
| L3.40-49 |  |  | .273*** | .239*** | .252*** | .171** | .133* | .096 | .084 | .283*** |  |
|  |  |  | (.077) | (.087) | (.088) | (.085) | (.075) | (.062) | (.06) | (.076) |  |
| L4.40-49 |  |  |  | .066 | .064 | .074 | -.019 | -.018 | -.018 | -.023 |  |
|  |  |  |  | (.08) | (.089) | (.085) | (.076) | (.063) | (.06) | (.058) |  |
| L5.40-49 |  |  |  |  | .002 | -.119 | -.133* | -.04 | -.042 | -.041 |  |
|  |  |  |  |  | (.08) | (.084) | (.076) | (.063) | (.06) | (.058) |  |
| L6.40-49 |  |  |  |  |  | .238*** | -.028 | .03 | .052 | .048 |  |
|  |  |  |  |  |  | (.077) | (.076) | (.063) | (.061) | (.059) |  |
| L7.40-49 |  |  |  |  |  |  | .506*** | .772*** | .791*** | .815*** |  |
|  |  |  |  |  |  |  | (.07) | (.063) | (.061) | (.059) |  |
| L8.40-49 |  |  |  |  |  |  |  | -.637*** | -.436*** | -.344*** |  |
|  |  |  |  |  |  |  |  | (.064) | (.078) | (.078) |  |
| L9.40-49 |  |  |  |  |  |  |  |  | -.281*** | -.137* |  |
|  |  |  |  |  |  |  |  |  | (.073) | (.08) |  |
| L10.40-49 |  |  |  |  |  |  |  |  |  | -.289*** |  |
|  |  |  |  |  |  |  |  |  |  | (.074) |  |
| L.10-19 | -.104** | -.038 | .009 | .01 | -.003 | -.032 | -.124* | -.123** | -.129** | -.112* |  |
|  | (.048) | (.07) | (.071) | (.072) | (.073) | (.07) | (.065) | (.059) | (.057) | (.057) |  |
| L2.10-19 |  | -.087 | -.133* | -.125 | -.131 | -.125 | -.076 | .027 | .053 | .037 |  |
|  |  | (.071) | (.078) | (.08) | (.08) | (.076) | (.068) | (.057) | (.06) | (.058) |  |
| L3.10-19 |  |  | .016 | -.002 | -.007 | -.009 | 0 | -.032 | .019 | .021 |  |
|  |  |  | (.071) | (.08) | (.081) | (.077) | (.068) | (.056) | (.055) | (.059) |  |
| L4.10-19 |  |  |  | .011 | -.052 | -.025 | -.009 | -.019 | -.029 | .01 |  |
|  |  |  |  | (.072) | (.08) | (.077) | (.068) | (.056) | (.054) | (.053) |  |
| L5.10-19 |  |  |  |  | .106 | 0 | .06 | .045 | .037 | .022 |  |
|  |  |  |  |  | (.073) | (.076) | (.068) | (.056) | (.054) | (.052) |  |
| L6.10-19 |  |  |  |  |  | .125* | .101 | .022 | .026 | .021 |  |
|  |  |  |  |  |  | (.071) | (.068) | (.057) | (.054) | (.052) |  |
| L7.10-19 |  |  |  |  |  |  | -.037 | -.065 | -.08 | -.076 |  |
|  |  |  |  |  |  |  | (.065) | (.057) | (.054) | (.053) |  |
| L8.10-19 |  |  |  |  |  |  |  | .106* | .12** | .09 |  |
|  |  |  |  |  |  |  |  | (.059) | (.06) | (.059) |  |
| L9.10-19 |  |  |  |  |  |  |  |  | -.053 | -.05 |  |
|  |  |  |  |  |  |  |  |  | (.057) | (.058) |  |
| L10.10-19 |  |  |  |  |  |  |  |  |  | .005 |  |
|  |  |  |  |  |  |  |  |  |  | (.058) |  |
| Constant | 11.138*** | 10.307*** | 7.991*** | 7.642*** | 6.939*** | 5.308** | 4.623** | 3.977** | 4.458** | 5.177*** |  |
|  | (2.437) | (2.524) | (2.523) | (2.588) | (2.634) | (2.531) | (2.262) | (1.885) | (1.818) | (1.78) |  |
| Observations | 258 | 257 | 256 | 255 | 254 | 253 | 252 | 251 | 250 | 249 |  |
| R-squared | .641 | .65 | .677 | .677 | .68 | .716 | .779 | .85 | .865 | .875 |  |
|  |  |  |  |  |  |  |  |  |  |  |  |
| **Granger tests** |  |  |  |  |  |  |  |  |  |  |  |
| F | 4.69 | 3.05 | 2.09 | 1.44 | 1.53 | 1.48 | 2.06 | 1.04 | 1.10 | 0.81 |  |
| Sig. | 0.031 | 0.049 | 0.101 | 0.220 | 0.181 | 0.187 | 0.048 | 0.405 | 0.363 | 0.615 |  |
| *Standard errors are in parentheses* | | | | | | | | | | | |
| **** p<.01, ** p<.05, * p<.1* | | | | | | | | | | | |

# Table S5. Results from prevalence studies in Spain. For readability purposes the values have been rounded to two decimal digits.

|  |  |  |  |  |  |  |  |  |
| --- | --- | --- | --- | --- | --- | --- | --- | --- |
| **Prevalence (Round)** | | | | | | | | |
| **Age group** | **Total population** | **(I)** | **(II)** | **(III)** | **(IV)** | **Estimated real cases** | **Cases detected** | **% Total detection** |
| **0-9** | 746311 | 2.64 | 2.74 | 3.07 | 6.55 | 48904.59 | 19216 | 39.29 |
| **10-19** | 835030 | 3.85 | 3.75 | 3.81 | 8.55 | 71414.54 | 37242 | 52.14 |
| **20-29** | 837118 | 4.59 | 4.54 | 4.73 | 10.1 | 84548.91 | 38815 | 45.9 |
| **30-39** | 1015386 | 4.39 | 4.61 | 4.53 | 9.16 | 93010.98 | 40598 | 43.64 |
| **40-49** | 1296794 | 5.5 | 5.65 | 5.6 | 10.21 | 132457.11 | 49334 | 37.24 |
| **50-59** | 1083699 | 5.84 | 5.95 | 5.94 | 11.39 | 123516.83 | 39532 | 32 |
| **60-69** | 832391 | 5.93 | 6.37 | 6.21 | 10.56 | 87969.43 | 24099 | 27.39 |
| **70-79** | 633302 | 6.21 | 6.82 | 6.52 | 10.62 | 67269.9 | 15857 | 23.57 |
| **80-89** | 354547 | 5.58 | 5.68 | 5.58 | 9.33 | 33110.22 | 12580 | 37.99 |
| **≥90** | 92451 | 5.4 | 7.6 | 7.7 | 8.5 | 7858.33 | 5370 | 68.33 |
|  |  |  |  |  |  |  |  |  |

Table S6. Detection rate at Catalonia, ^(1)^ the prevalence goes down in this second round due to the confidence interval and the small time between the two rounds, ^(2)^ shows the increment on the cases between the Round III and Round IV.

|  |  |  |  |  |  |
| --- | --- | --- | --- | --- | --- |
| **Catalunya** | **Round I** | **Round II** | **Round III** | **Round IV** | **Round IV-III^(2)^** |
| Prevalence | 5.8 | 6.1 | 5.90^(1)^ | 11.6 | 5.7 |
| Detected cases | 62360 | 67889 | 71057 | 334591 | 263534 |
| Real cases | 448167.68 | 471348.77 | 455894.71 | 896335.36 | 440440.65 |
| % Detection | 13.91 | 14.4 | 15.59 | 37.33 | 59.83 |
|  |  |  |  |  |  |

Table S7. Percent of detection for the Round III.

|  |  |  |  |  |  |
| --- | --- | --- | --- | --- | --- |
| **Age groups** | **Prevalence** | **Estimated**  **reals cases** | **Detected** | **% Detection** | **% Detection for Catalonia** |
| **0-9** | 3.07 | 22939.8 | 403 | 1.75 | 1.53 |
| **10-19** | 3.81 | 31830.4 | 731 | 2.29 | 2 |
| **20-29** | 4.73 | 39633.79 | 5416 | 13.66 | 11.91 |
| **30-39** | 4.53 | 45999.26 | 7223 | 15.7 | 13.69 |
| **40-49** | 5.6 | 72697.02 | 10618 | 14.6 | 12.73 |
| **50-59** | 5.94 | 64473.6 | 11500 | 17.83 | 15.55 |
| **60-69** | 6.21 | 51712.55 | 8635 | 16.69 | 14.56 |
| **70-79** | 6.52 | 41325.85 | 8343 | 20.18 | 17.6 |
| **80-89** | 5.58 | 19795.61 | 11186 | 56.5 | 49.27 |
| **≥90** | 7.7 | 7118.72 | 7002 | 98.36 | 85.76 |
|  |  |  |  |  |  |

Table S8. Percent of detection for the period IV-III by age group. ^(1)^ This is an error due to the sample size for this age group. Since we did not work with this age group, it does not affect our analysis.

|  |  |  |  |  |  |
| --- | --- | --- | --- | --- | --- |
| **Age groups** | **Prevalence** | **Reals** | **Detected** | **% Detection** | **% Detection for Catalonia** |
| **0-9** | 3.47 | 25964.78 | 18185 | 70.03 | 56.05 |
| **10-19** | 4.74 | 39584.14 | 34600 | 87.4 | 69.96 |
| **20-29** | 5.36 | 44915.12 | 36625 | 81.54 | 65,267 |
| **30-39** | 4.62 | 47011.72 | 38101 | 81.04 | 64.87 |
| **40-49** | 4.6 | 59760.08 | 46113 | 77.16 | 61.76 |
| **50-59** | 5.44 | 59043.23 | 36889 | 62.47 | 50 |
| **60-69** | 4.35 | 36256.88 | 22294 | 61.48 | 49.21 |
| **70-79** | 4.09 | 25944.05 | 14495 | 55.87 | 44.71 |
| **80-89** | 3.75 | 13314.61 | 11399 | 85.61 | 68.52 |
| **≥90** | 0.8 | 739.6 | 4833 | 653.45^(1)^ | 523.03^(1)^ |
|  |  |  |  |  |  |
